# Pre-anesthetic use of butorphanol for the prevention of emergence agitation in thoracic surgery: A multicenter, randomized controlled trial

**DOI:** 10.3389/fmed.2022.1040168

**Published:** 2022-12-13

**Authors:** Tao Meng, Xiaowen Lin, Ximing Li, Fangli Yue, Yuzhu Zhang, Yingbin Wang, Jianhua Gu, Zaiqi Yang, Hongli Yu, Kun Lv, Shengyong Liang, Xingda Li, Weibo Zhu, Gang Yu, Tao Li, Yujia Ren, Yandong Li, Jianjun Xu, Weimin Xu, Shu Wang, Jianbo Wu

**Affiliations:** ^1^Department of Anesthesiology, Qilu Hospital of Shandong University, Jinan, China; ^2^Department of Pain Management, Shandong Provincial Hospital Affiliated to Shandong First Medical University, Jinan, China; ^3^Department of Anesthesiology, Linyi People’s Hospital, Linyi, China; ^4^Department of Anesthesiology, Weifang People’s Hospital, Weifang, China; ^5^Department of Anesthesiology, Zibo Central Hospital, Zibo, China; ^6^Department of Anesthesiology, Lanzhou University Second Hospital, Lanzhou, China; ^7^Department of Anesthesiology, Jinan People’s Hospital, Jinan, China; ^8^Department of Anesthesiology, Taian City Central Hospital, Taian, China; ^9^Department of Anesthesiology, Tianjin First Central Hospital, Tianjin, China; ^10^Department of Anesthesiology, Jining No.1 People’s Hospital, Jining, China; ^11^Department of Anesthesiology, Feicheng People’s Hospital, Feicheng, China; ^12^Department of Anesthesiology, Linyi Central Hospital, Linyi, China; ^13^Department of Anesthesiology, Binzhou People’s Hospital, Binzhou, China; ^14^Department of Anesthesiology, Binzhou Central Hospital, Binzhou, China; ^15^Department of Anesthesiology, Yantai Yuhuangding Hospital, Yantai, China; ^16^Department of Anesthesiology, The Affiliated Hospital of Qingdao University, Qingdao, China; ^17^Department of Anesthesiology, Affiliated Hospital of Jining Medical University, Jining, China; ^18^Department of Anesthesiology, Daqing Oilfield General Hospital, Daqing, China; ^19^Department of Anesthesiology, Shengli Oilfield Central Hospital, Dongying, China; ^20^Department of Anesthesiology, Benxi Central Hospital, Benxi, China; ^21^Department of Anesthesiology and Perioperative Medicine, Qilu Hospital Dezhou Hospital, Shandong University, Dezhou, China

**Keywords:** butorphanol, emergence agitation, general anesthesia, thoracic surgery, single injection

## Abstract

**Background:**

Emergence agitation (EA) is common in patients after general anesthesia (GA) and is associated with poor outcomes. Patients with thoracic surgery have a higher incidence of EA compared with other surgery. This study aimed to investigate the impact of pre-anesthetic butorphanol infusion on the incidence of EA in patients undergoing thoracic surgery with GA.

**Materials and methods:**

This prospective randomized controlled trial (RCT) was conducted in 20 tertiary hospitals in China. A total of 668 patients undergoing elective video-assisted thoracoscopic lobectomy/segmentectomy for lung cancer were assessed for eligibility, and 620 patients were enrolled. In total, 296 patients who received butorphanol and 306 control patients were included in the intention-to-treat analysis. Patients in the intervention group received butorphanol 0.02 mg/kg 15 min before induction of anesthesia. Patients in the control group received volume-matched normal saline in the same schedule. The primary outcome was the incidence of EA after 5 min of extubation, and EA was evaluated using the Riker Sedation-Agitation Scale (RSAS). The incidence of EA was determined by the chi-square test, with a significance of *P* < 0.05.

**Results:**

In total, 296 patients who received butorphanol and 306 control patients were included in the intention-to-treat analysis. The incidence of EA 5 min after extubation was lower with butorphanol treatment: 9.8% (29 of 296) vs. 24.5% (75 of 306) in the control group (*P* = 0.0001). Patients who received butorphanol had a lower incidence of drug-related complications (including injecting propofol pain and coughing with sufentanil): 112 of 296 vs. 199 of 306 in the control group (*P* = 0.001) and 3 of 296 vs. 35 of 306 in the control group (*P* = 0.0001).

**Conclusion:**

The pre-anesthetic administration of butorphanol reduced the incidence of EA after thoracic surgery under GA.

**Clinical trial registration:**

[http://www.chictr.org.cn/showproj.aspx?proj=42684], identifier [ChiCTR1900025705].

## 1 Introduction

General anesthesia (GA) is the most widely used anesthesia method in various types of surgery, and it has been recognized that emergence agitation (EA) is a common, serious complication after GA (1–3). EA is an inappropriate behavior during the awakening period of anesthesia, manifested by coexisting excitement, disorientation, and some inappropriate behaviors (4, 5). In addition, EA can also increase the patient’s medical expenses and related unintended medical consequences such as falling out of bed, extravasation of intravenous fluids, and dehiscence (6). Strong associations are found between EA and the post-operative presence of drainage tubes. Patients undergoing thoracic surgery with greater surgical trauma and post-operative indwelling of the chest drainage tube present a higher incidence of EA compared with other surgeries (7). It is of great significance to prevent the occurrence of EA in patients undergoing thoracic surgery.

Earlier opioids were used to reduce the incidence of EA (8, 9), but those drugs might conduct the incidence of delayed recovery, vomiting, nausea, respiratory depression, and chest wall muscle rigidity. Butorphanol is a novel opioid receptor agonist, which possesses a high affinity for κ-receptor and partial activation of δ-receptor (10), and associated complications can be attenuated compared with conventional opioid agonists. Pharmacological research demonstrated that the administration of butorphanol had the effect of analgesia and relaxation (11), which might reduce the incidence of EA. Reports of pre-anesthetic application of butorphanol to treat EA have not been seen. Our hypothesis was that pre-anesthetic butorphanol singe injection would decrease EA during anesthesia recovery. We conducted this study on patients undergoing thoracic surgery because these patients had a higher incidence of EA and were at high risk of adverse outcomes when EA occurred (12).

## 2 Materials and methods

We conducted this pragmatic, randomized, allocation concealed, open-label, parallel group, multicenter trial at 20 hospitals in eastern China. The trial protocol and statistical analysis plan were designed by the trial investigators and are available online. The trial was sponsored by the Qilu Hospital of Shandong University; it was approved by the research ethics committee of Qilu Hospital of Shandong University and by the institutional review board at each participating center and registered with the Chinese Clinical Trial Registry (ChiCTR1900025705). Written consent was obtained from the patients, their next of kin, or a legal representative.

### 2.1 Patients and randomization

This study was performed between October 2019 and September 2021. A total of 668 patients undergoing elective video-assisted thoracoscopic lobectomy/segmentectomy for lung cancer were assessed for eligibility, and 620 patients were enrolled. The exclusion criteria were age >70 or <18 years, American Society of Anesthesiologists (ASA) physical status > III, body mass index > 30 kg/m^2^, and cardiac ejection fraction < 40%. Previous history of any psychological diseases, epilepsy, or Parkinson’s disease; visual, hearing, language, or other barriers that impeded communication and assessment of EA; history of traumatic brain injury or neurosurgery; severe hepatic dysfunction (Child–Pugh grade C); or renal failure (requirement for renal replacement therapy) were also excluded.

Patients were allocated randomly to the butorphanol group and the control group according to a computer-generated random number table, with a fixed block size of two according to a 1:1 ratio, and the allocation was sealed in an opaque envelope. The primary investigator or coinvestigator prospectively collected the laboratory results, radiology reports, and data on the clinical course. The surgeon, patients, attending anesthesiologists, data recorder, and analyzer were blinded to the group assignments. Butorphanol (0.02 mg/kg) was diluted by saline to 10 ml and injected slowly for 1 min.

### 2.2 Intraoperative management

Patients were monitored using an electrocardiogram, pulse oximetry, and non-invasive blood pressure (one measurement every 3 min) while entering the operation room. Butorphanol or saline was given intravenously 15 min before GA induction, and 5 min later, the Ramsay score was tested. A numerical rating scale (NRS: 0 = no pain, 10 = worst pain imaginable) was tested during the radial artery catheter placement. GA was conducted with sufentanil 0.4 μg⋅kg^–1^, propofol 1.5–2.0 mg⋅kg^–1^, and cis-atracurium 0.01–0.02 mg⋅kg^–1^; in this period, pain on injection of propofol and choking cough response with sufentanil were observed. One lung ventilation was performed with a bronchial blocker or double-lumen tube. Anesthesia was maintained with inhaled sevoflurane 1.5% in oxygen and remifentanil at 0.1–0.2 μg⋅kg^–1^⋅min^–1^. After GA, placing the patient in the proper position, a single anesthesiologist with significant ultrasound-guided regional anesthesia experience performed a paravertebral block. Thoracoscopic surgery was performed *via* a two-port technique. A 3 cm operation port and a 1.5 cm observation port were, respectively, made in the fifth intercostal space at the anterior axillary line and in the seventh or eighth intercostal space at the posterior axillary line. Two chest tubes were inserted into the two incisions for air leakage and drainage after surgery. Patients were extubated at the end of surgery when were fully awake and breathed adequately. EA was evaluated by Riker Sedation-Agitation Scale (RSAS) at 5 min after extubation. Then, they were transferred to the post-anesthesia care unit (PACU) for a 1 h observation period and sent back to the ward.

### 2.3 Post-operative analgesia protocol and rescue analgesic

After arrival at PACU 15 min later, patients were requested to evaluate pain at rest using NRS. If the NRS score was >5 at rest, sufentanil (5 μg) was administered intravenously as rescue analgesia. Then, all patients were connected to the PCA device. The PCA device consisted of 2 μg⋅kg^–1^ sufentanil to 100 ml and was programmed as follows: 0.5 ml⋅h^–1^ background rate, 3 ml bolus doses, and 5 min-lockout interval. In the ward, patients used PCA when the NRS score at rest was >5 or on their demand.

### 2.4 Outcome measurements

The primary outcome was the incidence of EA after 5 min of extubation. A score >4 at the time point was regarded as EA. The secondary outcomes were assessed: (1) Ramsay score at 5 min after using butorphanol, (2) NRS scores at radial artery catheter placed, (3) intravenously injecting propofol pain while anesthesia induction, (4) coughing with sufentanil injection, and (5) NRS scores at 5 min after extubation and 15 min after arrival PACU. Other outcomes were anesthetic consumption, rescue analgesia requirement, block-related complications, and post-operative adverse effects, such as pruritus, urinary retention, nausea, and vomiting.

### 2.5 Sample size

This study was controlled by placebo and tested for efficacy between the two groups. The incidence of EA was 6 ± 2% in the butorphanol group and 20 ± 3% in the control group. With the power set at 80% and a two-sided significance level at 0.05, 434 patients were required to detect a difference. Considering a loss to follow-up rate of approximately 20%, 544 patients were to be enrolled in the study.

### 2.6 Statistical analysis

Statistical analyses were performed using the SPSS statistical package, version 18.0 (SPSS Inc., Chicago, IL, USA). The measurement data were analyzed by analysis of variance and expressed as mean ± standard deviation, and the enumeration data were expressed as the number of cases. Continuous data with a normal distribution were compared using an independent-samples *t*-test, and continuous data with a non-normal distribution were compared using an independent-samples Mann–Whitney U-test. The incidence of EA was determined by the chi-square test, with a significance of *P* < 0.05.

## 3 Results

**FIGURE 1:**
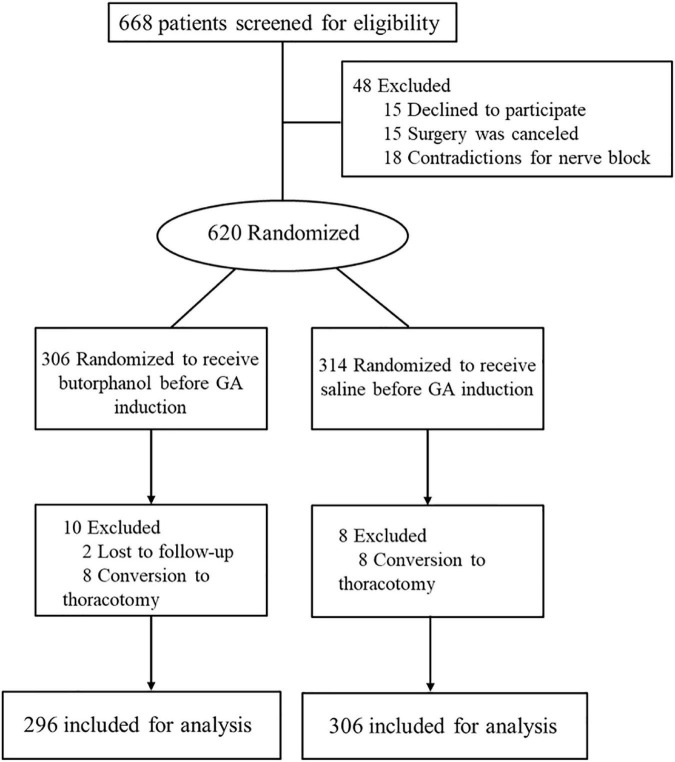
CONSORT diagram for the study.

From October 2019 to September 2021, 668 patients were screened for eligibility. Of those, 620 were enrolled and randomized (Figure 1). During the study, two patients were lost to follow-up and eight patients were converted to thoracotomy in each group. The baseline demographics and perioperative variables were similar in the two groups (Tables 1, 2). There was no significant difference in intraoperative anesthetic consumption.

**TABLE 1:** Baseline data.

	Butorphanol group (*n* = 296)	Control group (*n* = 306)
**Sex**		
Male	127 (42.9%)	122 (39.9%)
Female	169 (57.1%)	184 (60.1%)
Age (year)	54.15 ± 8.171	53.70 ± 8.816
Bodyweight (kg)	65.80 ± 10.209	64.42 ± 9.437
Height (cm)	165.05 ± 7.823	164.86 ± 7.275
BMI (kg/m^2^)	24.08 ± 2.683	23.62 ± 2.641
ASA grade	296 (100%)	306 (100%)
I	27 (9.1%)	17 (5.6%)
II	260 (87.8%)	276 (90.2)
III	9 (3.0%)	13 (4.2%)
Duration of surgery (min)	133.88 ± 103.92	127.74 ± 96.954

BMI, body mass index; ASA, American Society of Anesthesiologists. Data are presented as mean ± SD or number (%).

**TABLE 2:** Intraoperative data.

	Butorphanol group (*n* = 296)	Control group (*n* = 306)	*P*
Duration of anesthesia (h)	2.23 ± 1.73	2.13 ± 1.62	0.453
**Intraoperative drugs**			
Propofol (mg)	123.74 ± 29.02	117.15 ± 34.71	0.068
Sufentanil (μg)	27.56 ± 17.80	26.56 ± 6.35	0.357
Cis-atracuridinium (mg)	20.37 ± 7.12	20.11 ± 8.64	0.686
Remifentanil (mg)	1.64 ± 1.29	1.53 ± 1.08	0.368
Sevoflurane (ml)	27.73 ± 15.45	26.74 ± 18.38	0.478
SBP (mmHg)	134.07 ± 16.18	131.72 ± 16.12	0.074
DBP (mmHg)	80.17 ± 10.65	79.99 ± 10.24	0.83
Type of surgery			
Lobectomy	143 (51.3%)	136 (48.7%)	
Segmentectomy	153 (47.4%)	170 (52.6%)	

SBP, systolic blood pressure; DBP, diastolic blood pressure. Data are presented as mean ± SD or number (%).

**FIGURE 2:**
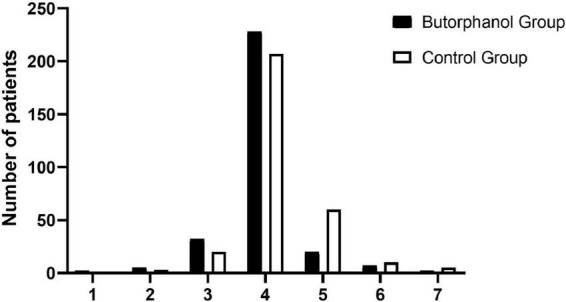
Distribution of the Riker Sedation-Agitation Scale (RSAS) at 5 min after extubation. 1, minimal or no response to noxious stimuli; 2, arousal to physical stimuli but non-communicative; 3, difficult to arouse but awakens to verbal stimuli or gentle shaking; 4, calm and follows commands; 5, anxious or physically agitated but calms to verbal instructions; 6, requires restraint and frequent verbal reminding of limits; and 7, attempting to remove tracheal tube, or catheters, or striking at staff.

Post-operative EA 5 min after extubation occurred in 29 of the 296 patients who received butorphanol and in 75 of the 306 patients in the control group (*P* = 0.0001) (Figure 2 and Table 3).

**TABLE 3:** Effectiveness outcomes.

	Butorphanol group (*n* = 296)	Control group (*n* = 306)	*P*	OR (95% CI)
**Primary outcome**				
Incidence of EA 5 min after extubation	29 (9.8%)	75 (24.5%)	0.0001	2.98 (1.88–4.75)
**Secondary outcomes**				
Ramsay score after using butorphanol >3	101 (34.1%)	22 (7.1%)	0.0001	6.69 (4.07–10.98)
**NRS > 5**				
Radial artery catheter placed	5 (1.7%)	44 (14.4%)	0.0001	0.1 (0.04–0.26)
5 min after extubation	18 (6.1%)	54 (17.7%)	0.03	3.31 (1.89–5.79)
15 min after arrival at PACU	23 (7.8%)	56 (18.3%)	0.03	2.54 (1.51–4.27)
Intravenously injecting propofol pain	112 (37.8%)	199 (65%)	0.001	3.06 (2.19–4.26)
Coughing with sufentanil injection	3 (1%)	35 (11.4%)	0.0001	12.61 (3.84–41.49)
Rescue sufentanil use	14 (4.7%)	23 (7.5%)	0.042	1.64 (0.83–3.25)

NRS, numerical rating scale. Data are presented as mean ± SD.

As indicated in Table 3, patients who received butorphanol had a lower incidence of drug-related complications (including injecting propofol pain and coughing with sufentanil): 112 of 296 vs. 199 of 306 in the control group (*P* = 0.001) and 3 of 296 vs. 35 of 306 in the control group (*P* = 0.0001). The NRS scores at the radial artery, placed 5 min after extubation and 15 min after arrival at PACU, were statistically significantly lower for the butorphanol group compared to the control group. The Ramsay scores after using butorphanol were different between the two groups (*P* = 0.0001). The rescue analgesia requirement in two groups showed that 14 of the 296 patients received butorphanol, and 23 of the 306 patients in the control group (*P* = 0.042).

## 4 Discussion

This study found that, in patients undergoing thoracoscopic lobectomy/segmentectomy surgery, pre-anesthetic butorphanol reduced the rate of post-operative EA. Butorphanol was associated with a lower rate of Ramsay score at 5 min after using butorphanol. Coughing with sufentanil injection and intravenously injecting propofol pain were also lower than in the control group.

In China, butorphanol, a common analgesic drug, is widely used for sedation and analgesia. It can be injected in a single dose before or during surgery, or be placed in an analgesic pump for post-operative analgesia. The strengths of the present study include the larger sample size than previous studies, multicenter study, and pre-anesthetic butorphanol single injection (13, 14). The larger sample size and multicenter study could increase the generalizability of the results. The mechanisms of the EA-sparing effect produced by pre-anesthetic butorphanol are still unclear, but it may be associated with the multiple effects of butorphanol such as calming effect, analgesic effect, and ease the tension before surgery (15–17). Butorphanol may attenuate the stress response provoked by surgery increasing the secretion of cortisol and hyper inflammation in the body (18), both of which are associated with an increased risk of EA.

For some drugs, lack of pre-operative loading dose is less effective in preventing post-operative EA in a few previous studies (19, 20). In line with previous reports, in our study, a single injection of butorphanol as a loading dose before anesthesia induction could release the pain of invasive manipulation, relieve the stress response and, finally, decrease the incidence of EA. These effects may be associated with reducing the side reactions of anesthetics during the induction phase such as infusion pain of propofol and cough reaction of sufentanil (21), and these may be related to activation of the μ-opioid receptor, as well as the competitive antagonist activity and partial agonist activity at the κ-opioid receptor, attenuate the side effects of propofol and sufentanil. With regard to safety, butorphanol (0.02 mg/kg) did not increase the incidence of side effects as opioid agonists, such as nausea, vomiting, delayed recovery, respiratory depression, and chest wall muscle rigidity before anesthesia and after surgery.

The control group had significantly more sufentanil consumption and higher VAS score than those in the butorphanol group 15 min after arrival at PACU. However, some doctors believed that they did not observe such a pronounced effect, the difference in sufentanil consumption might seem relatively small. Two major reasons may account for the results. First, it was probable that the good manipulation abilities and skills of surgeons caused less pain than expected. Second, anesthesiologists with different ultrasound-guided regional anesthesia experiences might perform different nerve block effects.

This study has several limitations. First, the primary outcome was EA, and the incidence of delirium for a long period after surgery should be observed. Second, as a multicenter study, anesthesia was administered by more than 40 different anesthesiologists, and this may include a diverse range of surgery and anesthesia qualities. Finally, because of the nature of the intervention, after using butorphanol, 34% of patients showed sedation before surgery, and blinding of treatment was not possible. However, the scoring of clinical outcomes was made by blinded assessors.

## 5 Conclusion

In conclusion, in patients undergoing thoracoscopic lobectomy/segmentectomy for lung cancer under GA, the pre-anesthetic administration of butorphanol reduces the incidence of EA after extubation. On the contrary, further studies are needed to determine the effect of the pre-anesthetic administration of butorphanol on long-term outcomes.

## Data availability statement

The original contributions presented in this study are included in the article/Supplementary material, further inquiries can be directed to the corresponding author.

## Ethics statement

The studies involving human participants were reviewed and approved by the Ethics Committee of Qilu Hospital of Shandong University. The patients/participants provided their written informed consent to participate in this study.

## Author contributions

All authors were involved in the study design, planning, study conduct, data analysis, revising, and drafting of the manuscript.
