# Supplementary figures and images for: Pre-anesthetic use of butorphanol for the prevention of emergence agitation in thoracic surgery: A multicenter, randomized controlled trial
Source: Front Med (Lausanne). 2022 Dec 13;9:1040168. doi: 10.3389/fmed.2022.1040168 (PMC9792474; doi:10.3389/fmed.2022.1040168)

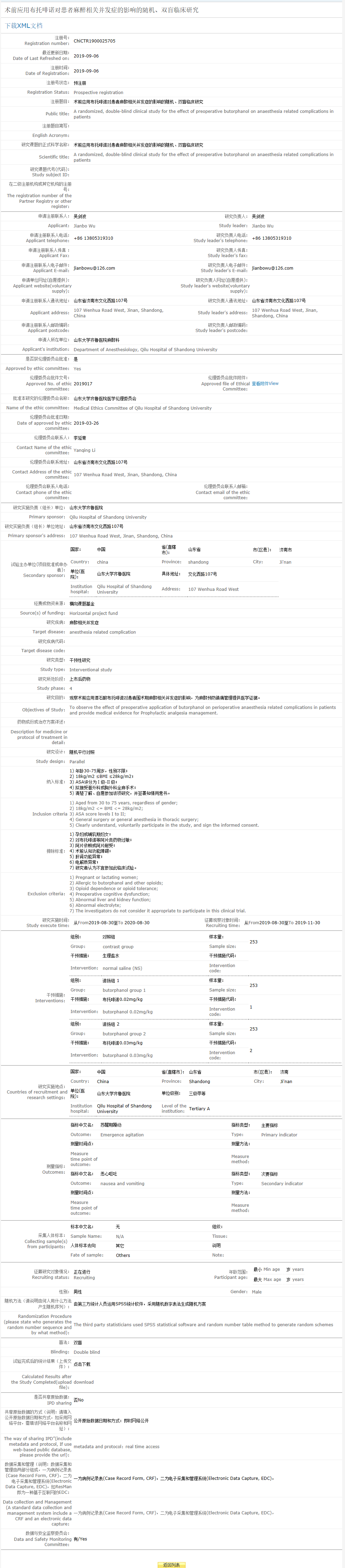

Supplement: Supplementary file 2 [file Image_1.PNG]
